# Effects of Drying on Viability of Germs

**Published:** 1916-07

**Authors:** 


**Effects of Drying on Viability of Germs.** Joseph M. Shapiro, Denver, Col. Med., May. Cholera 24 hrs., 16 days for 5-day-old culture, 42 days in moist chamber, 140 days after addition of bouillon (Ficker). 2 minutes on slide (Teague). Few hours (Buckley).

Typhoid. 4 and 9 days (Ficker). 1-5 days dried in sand, 4 days in faeces, 50 days on linen, 90 days on wool (Germano). 24 hrs. on Petri dishes (Kirstein). 36 hrs. on slides (Teague). 12 days on glass, 91 days on plaster (Buckley).

Diphtheria. 14 days in dessicator (Ficker). 30 days in dried sand, 50 days in moist sand, 4 months on membrane (Germano). 24-48 hrs. on Petri dishes (Kirstein). 60 hrs. (Teague). 6 days on paper, 41 days on lime wood (Buckley).

Colon Bacillus. 99% gone in 24 hrs. when dried in sand (Winslow and Abrahamson). 64 days on wood, 80 days on lime wood (Buckley).

B. Prodigiosus. 18 hrs. spraying on Petri dishes, 7 days spraying on silk, 68 days, washing silk in bacterial emulsion (Kirstein). 10 hrs. (Teague).

Staphylococcus. 8-10 days on Petri dishes (Kirstein). days (Teague). 53 days on glass, 140 days on plaster (Buckley).

Shapiro, after presenting these memoranda, described his experiments with diphtheria patients. He tested 6 swabs from a throat 5 weeks after the onset and 4 days after a positive diphtheria culture. No diphtheria bacilli were found but staphylococci were present. These were inoculated on to Petri dishes. Those exposed to indirect sunlight were all killed in less than 24 hours. Those in the shade died in 2-4

days. Two cases, seen some time after administration of antitoxin, yielded streptococci. Inoculated as before, these survived 2-4 days.

Employing a known culture of diphtheria bacilli, cotton swabs, glass rods and sticks of wood were inoculated and placed in glass containers with slightly lifted covers. Those on wood were dead by the 7th day, on glass by the 8th day, on cotton, by the 11th day. Various other experiments are detailed.

